# Supplementary material for: Pandemic H1N1 influenza A viruses suppress immunogenic RIPK3-driven dendritic cell death
Source: Nat Commun. 2017 Dec 5;8:1931. doi: 10.1038/s41467-017-02035-9 (PMC5715119; doi:10.1038/s41467-017-02035-9)
Supplement: Supplementary file 1 — Supplementary Information [file 41467_2017_2035_MOESM1_ESM.pdf]

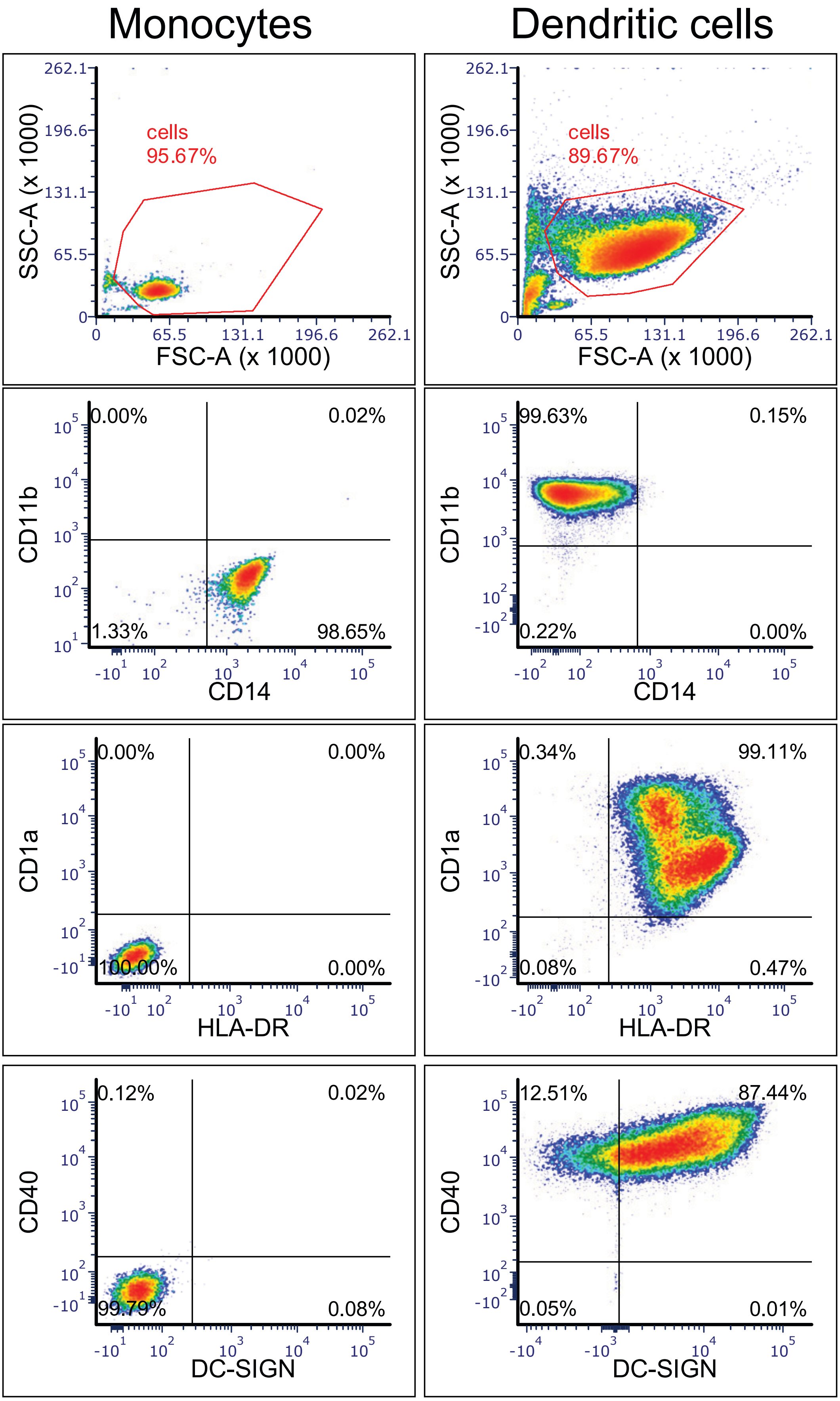

Supplementary Figure 1: Phenotyping of monocyte derived DC

CD14+ monocytes were differentiated into DC by culturing them for 5 days in the presence of IL4 and GM-CSF. Cells were phenotyped for CD11b, CD14, CD1a, CD40, DC-SIGN, and HLA-DR by flow cytometry before (left panels) and after (right panels) 5 days of culture.

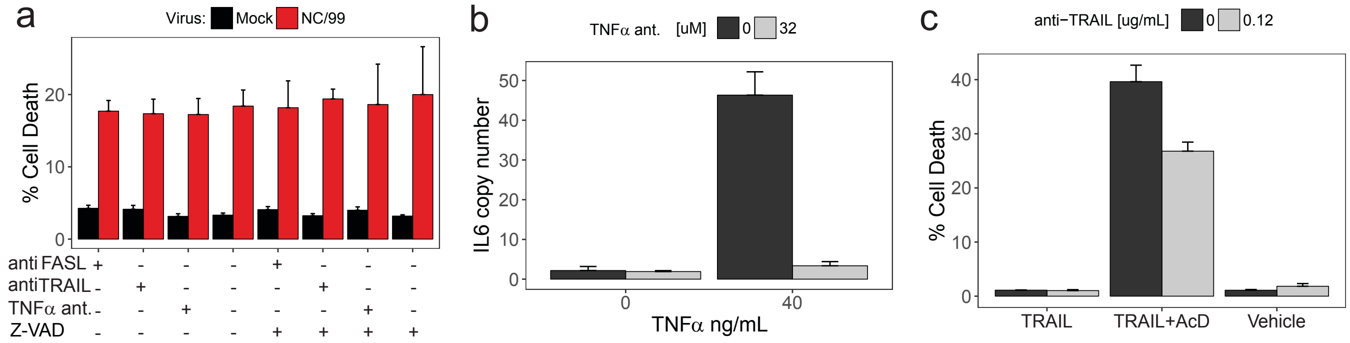

Supplementary Figure 2: Cell death induction by seasonal IAV is not mediated by major extrinsic pathway proteins.

(a) DC were infected with either mock or NC/99 IAV and cultured in the presence of anti-FASL or anti- TRAIL neutralizing antibodies, a TNFα antagonist, or pan-caspase inhibitor Z-VAD. (b,c) Control experiments showing the effectiveness of the TNFα antagonist and the anti-TRAIL neutralizing antibody in DC. Actinomycin D (AcD) was added to block the synthesis of anti-apoptotic proteins that may interfere with TRAIL-stimulated apoptosis. Values shown are median ± s.e.m..

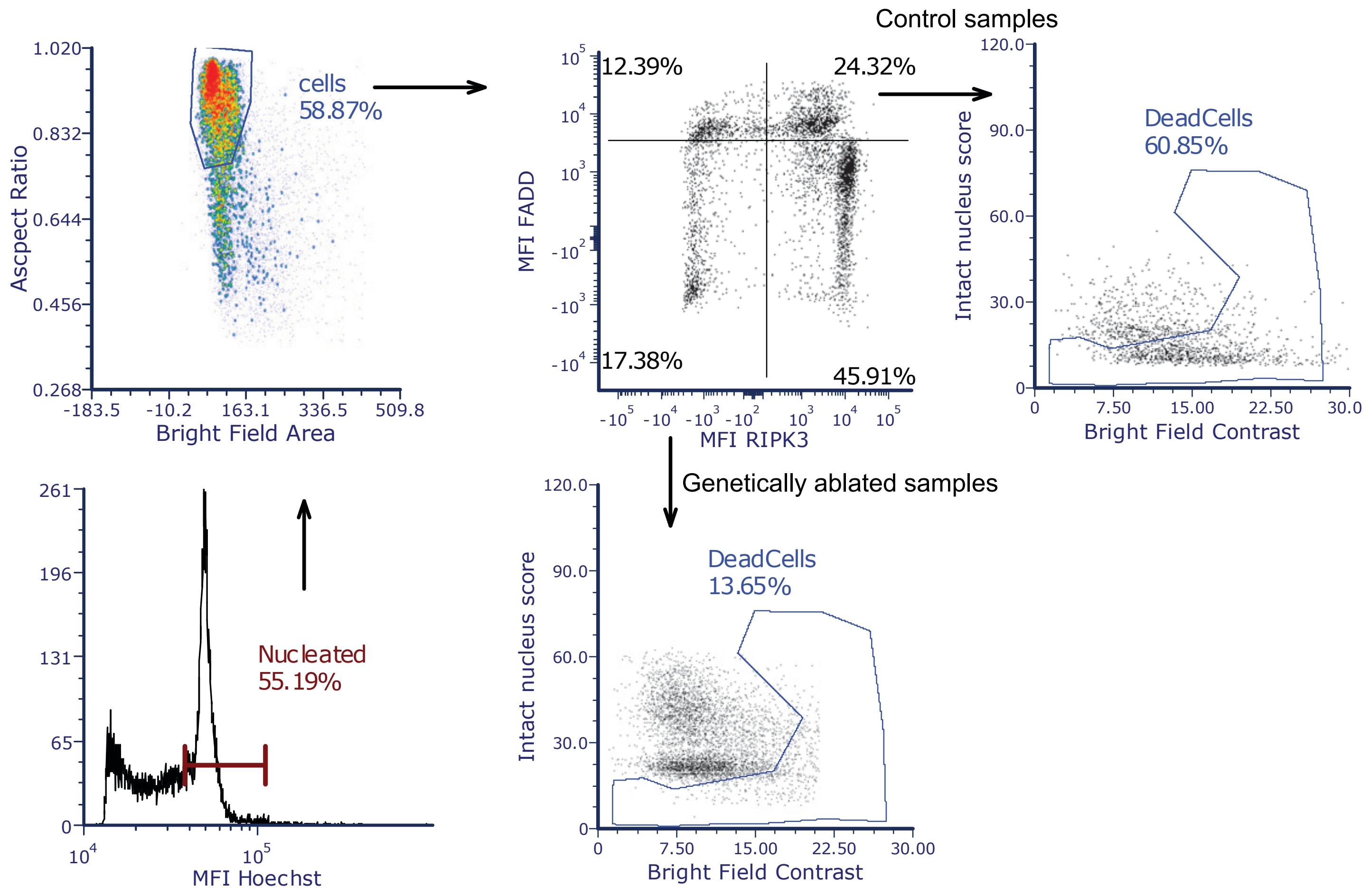

Supplementary Figure 3: Gating strategy for DC that have undergone genetic ablation with CRISPR/Cas9

Monocytes were electroporated with ribonucleoproteins consisting of nuclease Cas9 and short guide RNAs to target one or two specific genes. After 5 days of culture, DC were infected with NC/99 and fixed at 8 h post infection. Cells were permeabilized, stained for NP, stained with antibodies against the proteins encoded by the CRISPR-ablated genes, and with the nuclear dye Hoechst. Cells were initially gated on DNA staining and size, followed by a gate showing lower protein expression of the target gene(s) in the genetically ablated samples and a gate showing protein expression of the target gene(s) in the control samples.

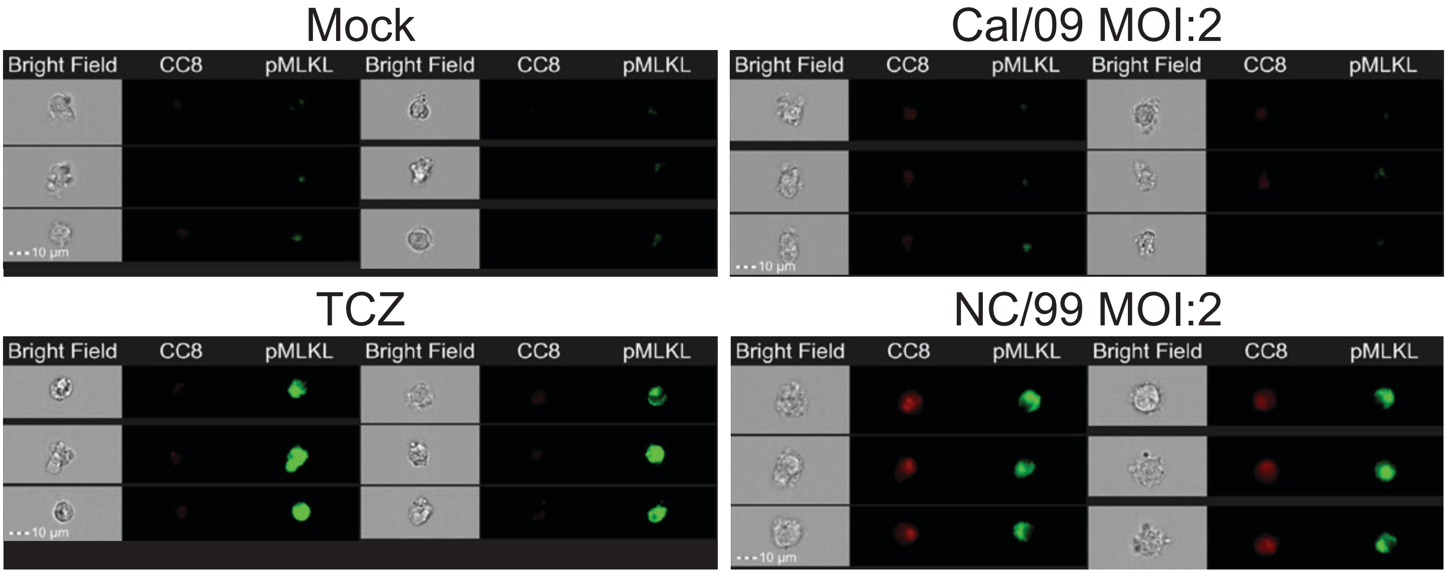

Supplementary Figure 4: Seasonal NC/99 IAV infection increases MLKL phosphorylation and caspase 8 cleavage in DC.

Sample raw imaging flow cytometry data for Fig. 3d. MLKL phosphorylation and caspase 8 cleavage were measured following infection with either mock, NC/99, or Cal/09 IAV at an MOI of 2. Also shown are the data obtained with TCZ-treated DC as a positive control for necroptosis. Shown are representative imaging flow cytometry plots from a replicate at 5 h post-infection or treatment.

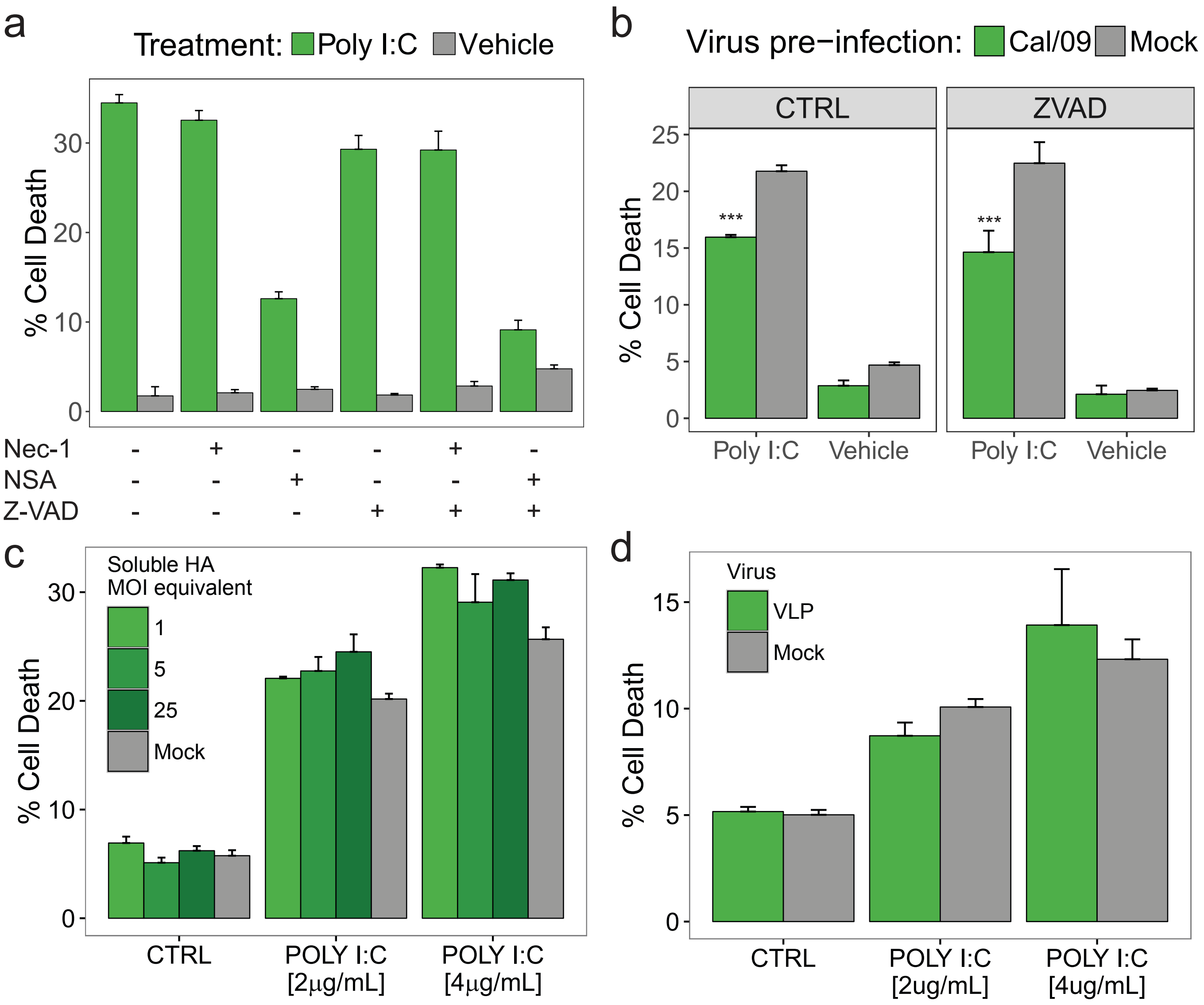

Supplementary Figure 5: Lack of effect of Nec-1, Z-VAD, soluble HA, and VLPs bearing HA on poly I:C-induced cell death

(a) DC were pretreated with the indicated pharmacological inhibitors for 1 h, then transfected for 6 h with either poly I:C (4 µg/ml; combined with the transfecting agent LYOVEC) or with vehicle. (b) Effect of Z-VAD pretreatment on Cal/09 inhibition of poly I:C-induced cell death. DC were infected with Cal/09 for 2 h in either the presence or absence of ZVAD, then transfected with poly I:C for 6 h. This experiment was performed 3 times. n=3 technical replicates. *** $p<0.005$, ANOVA followed by Tukey's HSD test. (c,d) DC were either exposed to soluble HA at MOI equivalents (c) or infected with virus-like particles (VLPs) bearing HA from Cal/09 (d) for 2 h, followed by poly I:C transfection for 6 h. Percentage of cell death was determined by imaging flow cytometry. Values shown are median ± s.e.m.

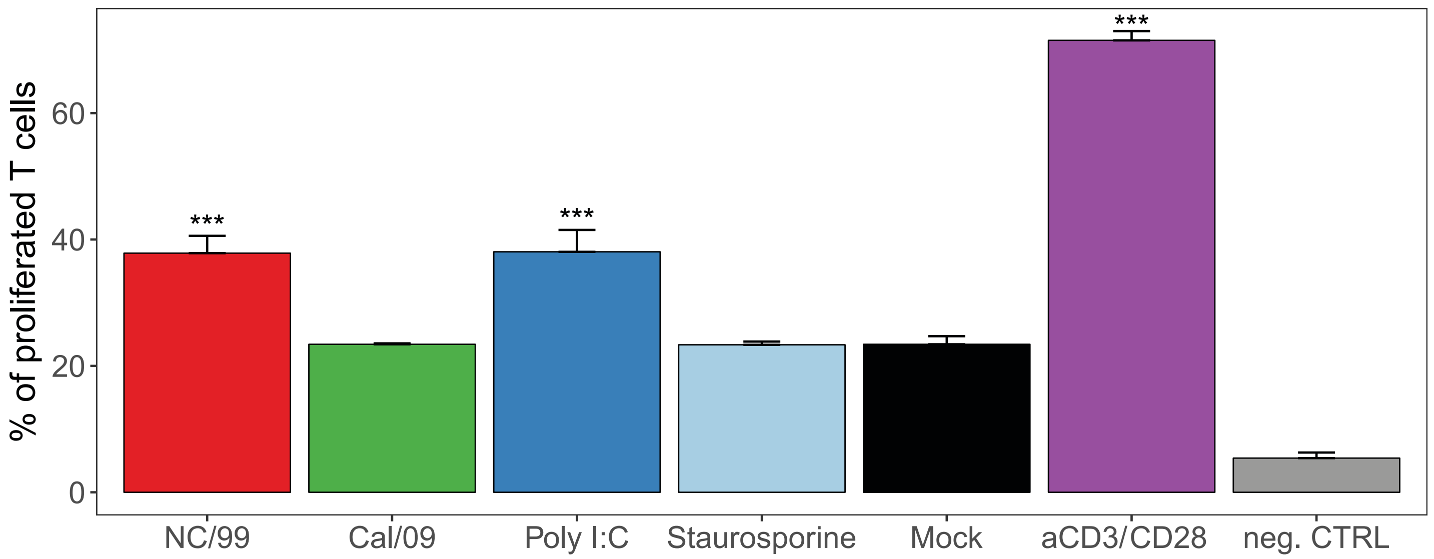

Supplementary Figure 6: Comparison of T cell proliferation induced by necroptotic vs. apoptotic DC

DC were either transfected with poly I:C for necroptosis induction or treated with staurosporine for apoptosis induction. Four hours after treatment, DC were co cultured with syngeneic DC for 4 h, and the DC mixture was exposed to allogeneic CFSE-labeled T cells for 5 days. Infection with necroptosis inducing NC/99, necroptosis-inhibiting Cal/09, or incubation of T cells with anti-CD3/CD28 beads served as controls. This experiment was performed 3 times. n=3 technical replicates. *** $p<0.005$, ANOVA followed by Tukey's HSD test. Values shown are median ± s.e.m.

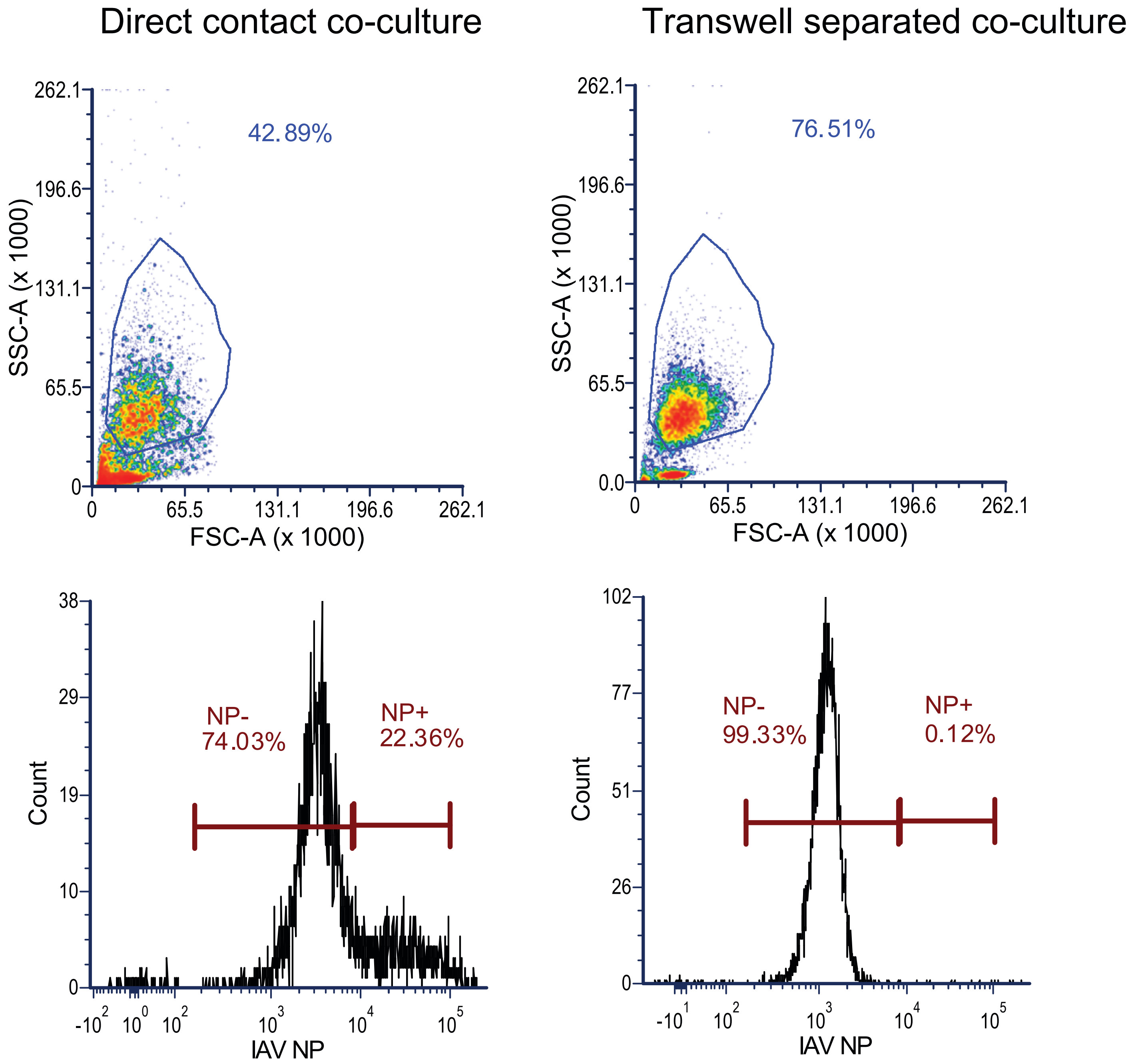

Supplementary Figure 7: Gating strategy for direct contact vs. transwell separated co-cultures of DC

DC were co-cultured with infected DC either by direct contact or via a transwell system. Uninfected DC in the direct contact co-culture were gated on negative expression of the viral NP protein. Although infected and uninfected cells were co-cultured at a 1:1 ratio, the lower proportion of NP-positive cells results from loss of infected cells at 18 h post infection. Because the lower chamber in the transwell system contains only uninfected DC, virtually no NP-positive cells are detected.

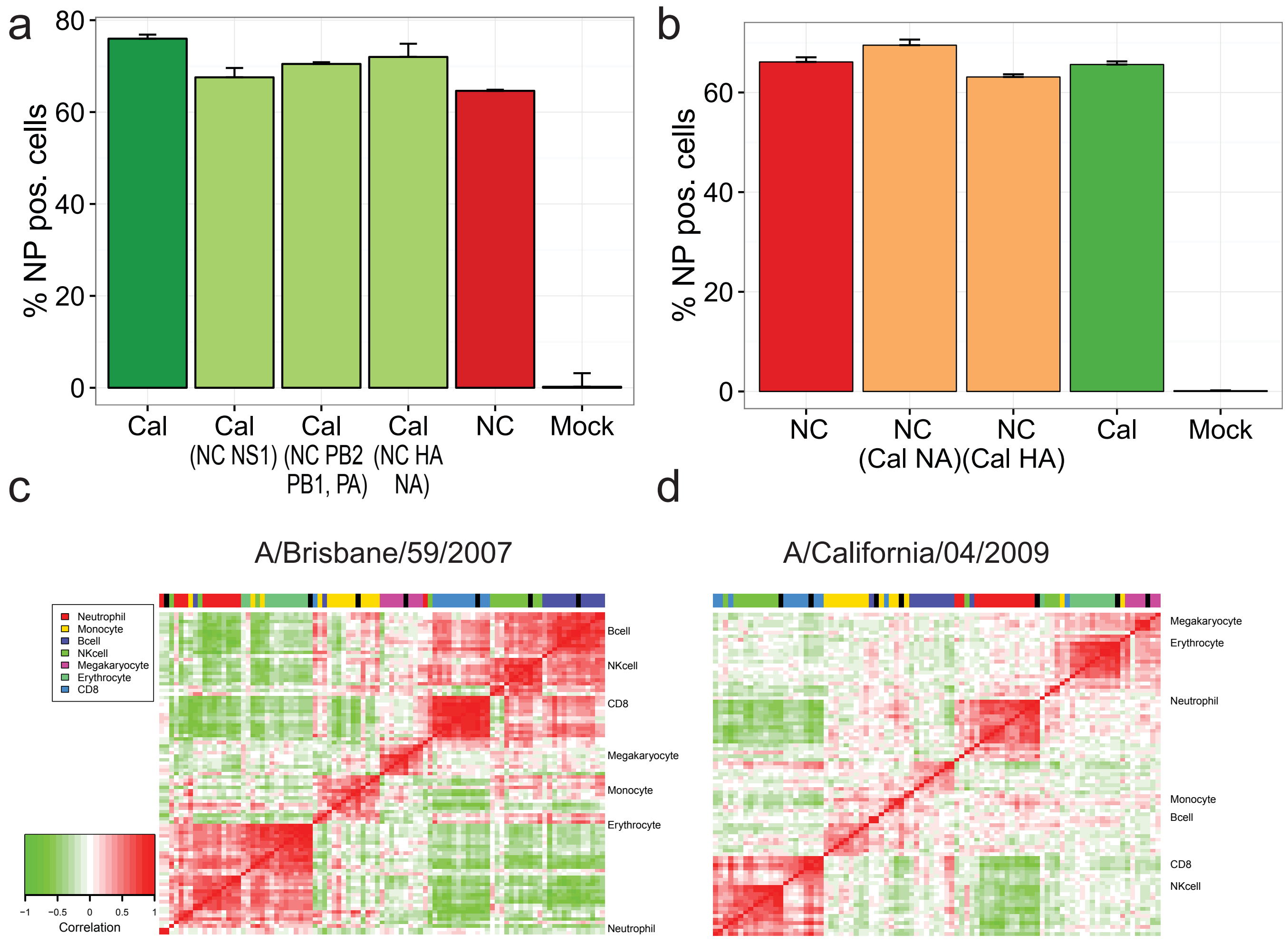

Supplementary Figure 8: Infectivity data of chimeric IAVs and blood cell type deconvolution matrix

(a,b) Infectivity data for Fig. 6a and Fig. 6b, respectively. Values shown are median ± s.e.m. (c,d) The reliability of cell proportion inference was assessed by inspecting a heat map of correlation coefficients for all pairs of cell type markers and surrogate proportion variables (SPVs) for each cell type (see Methods). Clustered heat maps for the seasonal A/Brisbane (c) and pandemic Cal/09 (d) are shown, with red representing high correlation and green representing anti-correlation. Marker genes initially selected for a specific cell type are indicated by colors, as shown in the key. The SPVs (indicated in black) are also included. Distinct clusters of high correlation emerge for each cell type, and each SPV reliably associates with the correct cluster in the analyses of both datasets.
